# Implementation of Multisetting Interventions to Address Childhood Obesity in Diverse, Lower-Income Communities: CDC’s Childhood Obesity Research Demonstration Projects

**DOI:** 10.5888/pcd14.170491

**Published:** 2017-12-21

**Authors:** Carrie A. Dooyema, Brook Belay, Heidi M. Blanck

**Affiliations:** 1 Division of Nutrition, Physical Activity, and Obesity, National Center for Chronic Disease and Health Promotion, Centers for Disease Control and Prevention, Atlanta Georgia; 2 US Public Health Service, Atlanta, Georgia

Childhood obesity continues to be a local, state, and national problem affecting not only children but their families, schools, employers, and communities. Obesity affects approximately 12.5 million (17%) US children and adolescents aged 2 to 19 years, with higher levels among some groups of children, including those living in low-income households. Obesity can have harmful effects during childhood. Children who have obesity are more likely to have high blood pressure and high cholesterol, which are risk factors for cardiovascular disease. They are more likely to have asthma, sleep apnea, fatty liver, insulin resistance, and type 2 diabetes. Obesity is also related to psychosocial problems in children, such as anxiety, depression, low self-esteem, and social problems such as bullying and stigma (1). To address obesity, the National Academy of Medicine (formerly the Institute of Medicine), among other groups, has called for interventions to alter nutrition and physical activity environments and promote behavior change in multiple settings to reach adults and children. For children, in addition to the home setting, other settings that can help support obesity prevention and aid healthy child growth include early care and education (ECE) or child care, schools, community, and health care (2).

In 2011, the Centers for Disease Control and Prevention’s (CDC’s) Division of Nutrition, Physical Activity, and Obesity funded 3 grantees under the 4-year Childhood Obesity Demonstration (CORD) Project. The 3 grantees are located in Massachusetts (MA CORD), California (CA CORD), and Texas (TX CORD). The aim of CORD was to improve weight and healthy growth among low-income children by improving obesity-related behaviors, including diet, physical activity, screen time, and sleep. Grantees engaged with community coalitions and organizations to deliver evidence-based interventions in the places where families live, learn, and seek health care, and they used the Obesity Chronic Care Model (3). The framework and research design of CORD are described elsewhere (4). The MA CORD project was conducted in 2 cities, one with approximately 40,000 residents and the other with approximately 95,000 residents. CA CORD took place in 3 rural communities along the California–Mexico border, and the TX CORD covered catchment areas in 2 large cities.

This special collection features 5 articles authored by CORD grantees and focuses on the real-world implementation of evidence-based interventions across multiple settings (5–9). CORD built on each community’s existing work and aimed to improve the knowledge and skills of parents, providers, teachers, and organizational leaders in nutrition, physical activity, and obesity. The collection explores and identifies factors that are critical to stakeholder engagement and implementation of interventions in racially and ethnically diverse communities.

The collection also helps highlight the importance of implementation science. The National Cancer Institute defines implementation science as the “study of methods to promote the adoption and integration of evidence-based practices, interventions and policies into routine health care and public health settings in order to improve our impact on population health” (10). This collection helps further our understanding of how interventions are adopted and integrated into existing organizations such as schools, health care facilities, and child care centers and delves into the factors necessary to build support and engagement for successful implementation. The collection can help local health departments, researchers, organization leaders, and community coalitions plan for and integrate evidence-based prevention and lifestyle-management interventions into routine settings for all children, by describing not only what to do but how to do it.

## Overview of articles in the collection

The article by Ganter et al (5), CORD researchers in Massachusetts, examines the role of stakeholder engagement to support the implementation of the multisetting CORD intervention and uses qualitative methods to identify successes and lessons learned. It offers insight into whole-of-community interventions and helps us understand the need for administrative and leadership support, early involvement of intervention implementers, and the importance of regular communication, especially across the intervention sectors. Researchers cite some of the successes of the MA CORD implementation, including high levels of acceptability of the intervention among target audiences, increased linkages to community resources, and opportunities to implement new intervention activities to benefit children and families in their community. Stakeholders also reported that increased engagement of parents was a vital feature associated with health care visits to primary care providers and providers in the Special Supplemental Nutrition Program for Women, Infants, and Children program. Parent engagement also improved participation rates in school activities. Stakeholders posited that improvements could have resulted from MA CORD’s consistent messaging to parents and families about 5 critical health behaviors along with increased community awareness of the problem of childhood obesity.

Researchers in CA CORD, Chuang et al (6), examined factors affecting implementation of the CA CORD intervention. They interviewed stakeholders and project leaders across each of the 3 rural CORD settings (school, ECE, and health care) and found similar implementation facilitators and barriers across the settings. Facilitators included engaging parents and obtaining support from all levels of the organization, including higher levels of organization leadership and key staff members, such as teachers who carried out intervention activities. Reported barriers included staff turnover and limited access to supportive resources in the community at large. Addressing these barriers may be particularly important in rural communities like those in CA CORD.

Byrd-Williams et al (7) help further our understanding of the perspectives of ECE providers such as directors and teachers. Their cross-sectional study elucidates how Head Start directors and teachers were meeting best practices for nutrition and physical activity and the barriers these important caregivers faced in implementing best practices. Common barriers such as lack of time, resources, and funds were cited by both teachers and directors. Public health practitioners may consider addressing these barriers when planning and implementing evidence-based ECE interventions.

CDC’s *School Health Guidelines to Promote Healthy Eating and Physical Activity* recommends comprehensive school interventions that have an impact on both nutrition and physical activity (11). However, more can be learned about how stakeholders in schools can increase capacity to undertake comprehensive interventions. The article by Blaine et al (8) focuses on the school setting. It uses a mixed-methods approach to describe facilitators and barriers to implementation in the 2 school districts in Massachusetts that participated in the CORD intervention. Facilitators included having the principal as a champion, using students as peers to engage other students, and integrating school-wide messaging strategies. Barriers included competing needs from standardized testing and academic requirements, teachers not being informed about the intervention, and staff turnover. The authors outline 4 essential lessons that may be helpful to researchers and practitioners in carrying out school-based interventions.

Finally, an article by Barlow et al (9) describes the real-world experiences in the health care setting related to recruitment and enrollment of low-income children with obesity from primary care practices into an intensive childhood obesity intervention based in the community. This descriptive analysis provides insight into what factors might cause providers to refer children to behavioral weight-management programs such as those in TX CORD and, more importantly, what factors influence families to enroll in these programs. Information in this article can help inform others about what strategies might be effective for recruiting children in low-income families into family-centered childhood weight-management programs.

This collection sheds light on factors affecting the implementation of multisector interventions or whole-of-community interventions, including what resonates with diverse stakeholders. These articles contribute to knowledge about how to effectively coordinate and implement approaches that aim to prevent childhood obesity and support children and families in diverse communities already struggling with obesity.
